# Behavioral Effects of 4-CMC and 4-MeO-PVP in DBA/2J Mice After Acute and Intermittent Administration and Following Withdrawal from Intermittent 14-Day Treatment

**DOI:** 10.1007/s12640-021-00329-x

**Published:** 2021-01-11

**Authors:** Jakub Wojcieszak, Katarzyna Kuczyńska, Jolanta B. Zawilska

**Affiliations:** Department of Pharmacodynamics, Medical University of Lodz, 90-151 Lodz, Poland

**Keywords:** Spontaneous locomotor activity, Sensitization, Conditioned place preference, Depression, Synthetic cathinones

## Abstract

Synthetic cathinones appeared on the market in the 2000s as new psychoactive substances and gained significant prevalence among drug abusers. Cathinones produce psychostimulant and empathogenic effects by enhancing dopaminergic, noradrenergic, and serotoninergic neurotransmission in the brain, and those which potently and selectively enhance dopaminergic transmission are considered to have higher abuse potential. The present study examines the behavioral effects related to psychostimulant properties, abuse potential, and addiction in DBA/2J mice of two cathinones with different profile of action on monoamine system, 4-chloromethcathinone (4-CMC), and 4-methoxy-pyrrolidinopentiophenone (4-MeO-PVP). 4-CMC and 4-MeO-PVP increase spontaneous locomotor activity after acute treatment and produce behavioral sensitization after 7-day intermittent treatment, which is a common feature of drugs of abuse. 4-MeO-PVP, but not 4-CMC, produces conditioned place preference after 4 days, indicating its rewarding properties. Finally, the ability of 4-CMC and 4-MeO-PVP to induce withdrawal symptoms after discontinuation from 14-day treatment was assessed using a battery of tests for behavioral markers of depression in mice: a tail suspension test, a forced swim test, measuring despair, and a sucrose preference test, measuring anhedonia. None of the three tests revealed increased depressive symptoms. Moreover, neither spontaneous locomotor activity nor motor performance on a rotarod was impaired after 14-day treatment with the tested compounds. These results indicate that 14-day treatment of mice with 4-CMC or 4-MeO-PVP does not induce significant withdrawal symptoms after cessation, nor significant impairment of dopaminergic circuitry resulting in motor impairment. The current study shows that 4-CMC and 4-MeO-PVP produce abuse-related behavioral changes in mice, which are more pronounced after more dopamine-selective 4-MeO-PVP.

## Introduction

Within the last two decades, we have witnessed the emergence and widespread abuse of new psychoactive substances (NPS), a broad group of recreational drugs introduced into the market in order to circumvent legal restrictions against classic drugs of abuse. Synthetic cathinone derivatives form one of the most prominent groups of NPS. They produce similar effects in humans to those observed after cocaine, methamphetamine or MDMA, mediated by stimulation of monoaminergic transmission in the central nervous system (EMCDDA 2019; Zawilska and Wojcieszak 2013). All NPS groups are characterized by a form of “arms race”: continual changes in the scheduling of particular substances by governments result in new analogs obtained by simple structural modifications being introduced into the market. As a result, when popular synthetic cathinones such as mephedrone and MDPV were banned, numerous new analogs were seized by authorities and characterized in forensic laboratories. Among the new derivatives, 4-chloromethcathinone (4-CMC; clephedrone) and 4-methoxy-pyrrolidinopentiophenone (4-MeO-PVP) were detected in seized products in Europe and Japan (Białas et al. 2017; Ellefsen et al. 2016; EMCDDA 2016, 2018; Taschwer et al. 2014; Uchiyama et al. 2013). Although 4-CMC and 4-MeO-PVP bear the same core structure of β-cathinone, they show subtle differences in their molecular mechanisms of action. According to Eshleman and colleagues (2017), 4-MeO-PVP is a monoamine uptake inhibitor with a significant preference for dopamine transporter (DAT) over serotonin transporter (SERT). Its potency for uptake inhibition (IC_50_) was found to be 0.1126 µM for DAT and 4.18 µM for SERT, resulting in a DAT/SERT inhibition ratio of 37.1. In contrast, 4-CMC is rather equipotent for monoamine transporters (IC_50_: 0.208 µM for DAT and 0.67 µM for SERT) with a DAT/SERT inhibition ratio of 3.22 (Eshleman et al. 2017). Importantly, 4-CMC, in contrast to 4-MeO-PVP, is also a potent DA and 5-HT releaser (Bonano et al. 2015; Eshleman et al. 2017). The profile of action on monoamine transporters is widely accepted as an indicator of expected abuse potential. Drugs selectively enhancing DA-ergic and NA-ergic transmissions are usually endowed with high addictive properties mediated by profound stimulation of the reward system, while compounds which also potently stimulate serotoninergic transmission are more likely to have lesser abuse potential and are usually considered as “weekend” or “club” drugs (Liechti 2015; Zawilska and Wojcieszak 2013).

The aim of the present study was to assess the behavioral effects of 4-CMC and 4-MeO-PVP in mice, with a special emphasis given to behavioral markers related to addiction and psychostimulant properties, such as the ability to stimulate spontaneous locomotor activity after acute treatment and to produce behavioral sensitization and place preference after repeated administration. It also tested whether signs of depression or dopaminergic toxicity would occur during withdrawal after prolonged 14-day treatment schedule.

## Materials and Methods

### Animals and Treatment

For all experiments, male DBA/2J mice (Mossakowski Medical Research Center Polish Academy of Sciences, Warsaw) at 10–12 weeks of age were used. Selection of a mouse strain was based on published data indicating low basal immobility of DBA/2J mice in forced swim and tail suspension tests, allowing for more sensitive detection of potential depressive effects, and its sensitivity to acute desipramine and fluoxetine as well as chronic lithium treatment (Can et al. 2011; Lucki et al. 2001). Moreover, the behavioral characteristics of DBA/2J mice differ markedly from previously used C57BL/6J (Võikar et al. 2005; Wojcieszak et al. 2020). Therefore, it was used to confirm whether previous findings regarding the effects of 4-CMC and 4-MeO-PVP are independent of genetic background.

All housing conditions and experimental procedures were in accordance with the European Union guidelines regarding the care and use of laboratory animals (European Communities Council Directive of September 2010 (2010/63/EU)) and ARRIVE guidelines and were approved by the Local Ethical Commission for Experimentations on Animals in Łódź. Mice were housed in groups of 2–4 per standard cage (cage height 14 cm, minimal surface/mouse = 132.5 cm^2^), enriched with one tunnel, with ad libitum access to tap water and standard chow. Cages were kept in a sound-attenuated room with automatic 12-h light/dark cycles (lights on at 6:00 a.m.), at temperature of 20–24 °C and relative humidity of 45–65%. Upon arrival, the animals were allowed to acclimatize to the new facility for 1 week. The study consisted of four experiments, with separate cohorts of mice used for each experiment. Every treatment was delivered by a subcutaneous injection; control groups were always injected with 0.9% NaCl, while 4-CMC and 4-MeO-PVP groups were treated with drug solutions prepared in isotonic saline. All injections were delivered at a volume of 0.1 ml/10 g body mass. In a case of repeated treatments, a site of injection was cyclically changed in order to avoid irritation and tissue damage. All treatments and experimental procedures were conducted during the light phase (approx. 8:00 a.m.–3:00 p.m.). When animals belonging to different treatment groups had to be tested during the same day, the order of testing was counterbalanced to avoid influencing results by circadian rhythm-based changes in rodent activity.

## Drugs

4-CMC (4-chloromethcathinone; clephedrone; 1-(4-chlorophenyl)-2-(methylamino)-1-propanone) and 4-MeO-PVP (4-methoxy-α-pyrrolidinopentiophenone; 1-(4-methoxyphenyl)-2-(1-pyrrolidinyl)-1-pentanone) were purchased as hydrochloride salts from Cayman Chemical (Ann Arbor, MI, USA). Isotonic saline solution for injections was purchased from Polska Grupa Farmaceutyczna (Łódź, Poland).

### Experiment 1—Effects of Acute Treatment on the Spontaneous Locomotor Activity

The acute effects of 4-CMC and 4-MeO-PVP on the spontaneous activity of mice were first assessed to select the drug doses to be used in later studies requiring multiple treatments. Spontaneous locomotor activity was measured according to Wojcieszak et al. (2020) with minor modifications, using Opto-Varimex Auto-Track (model 0271-002M, Columbus Instruments, Columbus, OH, USA) open field chambers (20.3 × 20.3 × 20.3 cm; 4 units) equipped with 16 infrared beams and corresponding photodetectors, spaced by 1.3 cm; these were located on the X and Y horizontal axes, on two planes, to detect both horizontal and vertical movements.

The mice were randomly assigned to treatment groups, consisting of seven (4-CMC 5 mg/kg; one mouse was killed by cage mates) or eight mice: control (0.9% saline), 4-CMC (10, 20 mg/kg) and 4-MeO-PVP (5, 10, 20 mg/kg). The day before the measurement, the animals were allowed to habituate to the apparatus for 60 min. On the test day, mice were allowed to further habituate for 30 min, after which injections were delivered and the animals were immediately placed into the chamber for measurement of locomotor activity for 120 min. Both habituation and measurement were conducted under a dim red light. Experimental analysis was based on counts of beam breaks on the bottom and top layers within 10-min intervals. Beam breaks were recorded every 0.1 s and based on their sequence; software provided with Opto-Varimex Auto-Track system tracked the position (X, Y, Z) of the animal and calculated parameters related to its movement, such as path, speed, and distance covered. Stereotypies, defined as repetitive movements, were automatically detected using a parameter called “fence size” (set to 2.5 cm): if an animal performed a repetitive movement within this range and did not move beyond it within 1 s, it was regarded as the stereotypic movement. This accounts for small movements without changing the position of the animal.

### Experiment 2—Ability to Produce Behavioral Sensitization

During this set of experiments, animals were divided into five experimental groups consisting of eight mice each: control (0.9% saline), 4-CMC (5 and 10 mg/kg), and 4-MeO-PVP (5 and 10 mg/kg). The 20-mg/kg dose was omitted because in the acute experiment, 4-CMC produced delayed locomotor stimulation (probably due to increased stereotypies in some mice, see Supplementary Material 1), while 4-MeO-PVP induced very high stimulation. In light of these observations, there was a significant risk that following an intermittent treatment, stereotypies would be more likely to occur in some mice, thus impeding a clear interpretation of data (Aarde et al. 2013). The animals were injected once daily during the first 7 days. After 10 days of abstinence, the mice were challenged with the same treatment on the 18th day. Spontaneous locomotor activity was measured as in the experiment 1 on days 1, 7, and 18. The total distance covered by mice during each 120-min experimental session was considered an index of the drug response potency and subjected to statistical analysis. Sensitization was considered when there was a significant increase of distance covered during 120 min in response to drug treatment between consecutive sessions.

### Experiment 3—Rewarding Properties (Conditioned Place Preference; CPP)

The rewarding properties of 4-CMC and 4-MeO-PVP were assessed using a biased CPP paradigm. In brief, animals were divided into five groups: control (0.9% NaCl), 4-CMC (10 mg/kg and 20 mg/kg), and 4-MeO-PVP (10 mg/kg and 20 mg/kg), each consisting of 10 mice, except of 4-MeO-PVP 20 mg/kg (9 mice). The apparatus consisted of two chambers [20 (length) × 20 (width) × 30 (height) cm] separated by a wall with removable guillotine doors. Chambers were visually and tactilely distinct: one had a smooth white floor and walls with vertical black/white stripes, while another one had textured floor (5 × 5 mm tiles with 1 mm of height difference, arranged as on the chessboard) and plain black walls. Before the start of the experiment, the mice were handled once daily for 1 week and introduced once to the testing room to reduce stress. On the first day, the mice were allowed to explore the apparatus with doors removed for 20 min (pre-test). This stage was recorded with digital cameras located above the apparatus and manually scored by two experimenters with the aid of self-developed software (Supplementary Material 2) to assess initial preferences. On days 2–5, each mouse was subjected to two conditioning sessions, each lasting for 40 min. The first session started at 8:00 a.m.: each mouse obtained saline injection and was placed immediately to initially preferred side of apparatus (doors closed). During the second session, which started after a 4-h break, each mouse obtained either saline (control group) or a drug injection and was placed in the initially non-preferred compartment (doors closed).

Conditioning sessions were not recorded. Post-test was conducted on the day 6, with the same procedure as the pre-test on day 1. During scoring, experimenters were blind to the treatment, and mouse was considered to be in a compartment when the head and both forelimbs were inside. The exclusion criteria were as follows: > 80% of time spent in one compartment and < 6 crossings between compartments on day 1. One mouse had to be excluded. The initial preference was on a similar level (% of total time spent in chambers: black 45.82%, striped 54.18%). All sessions were carried out under a dim white dispersed light (portable lamp directed at the room floor) to prevent uneven illumination of the chambers. After each session, the chambers were thoroughly cleaned with isopropyl alcohol to remove olfactory cues, and each mouse was always put in the same chamber, placed in the same location to provide additional distal clues. The length of testing and conditioning sessions was selected based on (a) the onset and duration of drug action observed in Experiment 1 (see Fig. 2 and Supplementary Material 1), (b) the length of conditioning sessions which was previously found to allow detection of cocaine-induced place preference in DBA/2J mice (Cunningham et al. 1999; Orsini et al. 2005). The drug treatment was always performed during the second daily session to exclude the possibility that the saline-paired session would be performed when the drug effects were wearing off (approx. 4 h after injection), which could produce an association with unpleasant (aversive) stimulus. A preference index was calculated as a difference between time spent in drug-paired (initially non-preferred) chamber during post- and pre-tests.

### Experiment 4—Withdrawal Effects

**Fig. 1:**
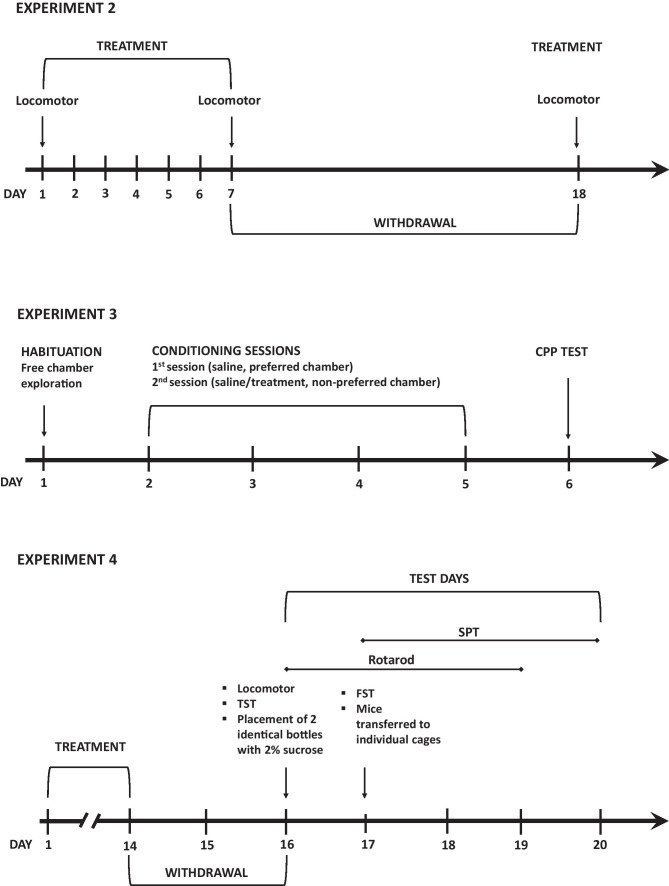
Schematic representation of treatment schedules and order of testing in experiments 2, 3, and 4. CPP–conditioned place preference, FST–forced swim test, SPT–sucrose preference test, TST–tail suspension test

This experiment tested whether the 14-day intermittent treatment of mice with 4-CMC and 4-MeO-PVP, both at 10 mg/kg, followed by 48 h of abstinence could evoke withdrawal symptoms. A group size was 12 mice, with a random assignment. The dose of 10 mg/kg was selected as it produced significant increases of locomotor activity and sensitization for both drugs and evoked CPP for 4-MeO-PVP. Schematic presentation of Experiments 2, 3, and 4 is pictured in Fig. 1.

#### Spontaneous Locomotor Activity

The effects of 4-CMC and 4-MeO-PVP withdrawal on the spontaneous locomotor activity of mice were measured approx. 48 h after the last injection of the drugs. Locomotor activity was assessed during a 30-min trial for each mouse.

#### Behavioral Markers of Depression

To assess the possible pro-depressive effects of drug withdrawal, two behavioral markers were analyzed: (a) despair, using the forced swim test (FST), and tail suspension tests (TST) according to standard protocols (Can et al. 2012, Castagné et al. 2011), and (b) anhedonia using a sucrose preference test (SPT).

#### Tail Suspension Test

The first test for despair was conducted approximately 1 h after the completion of measurement of spontaneous locomotor activity. Briefly, mice were suspended by their tails (approx. 1 cm from the tips) using adhesive tape to the horizontal bars and their behavior was recorded for 6 min. A distance from the nose to the ground was approx. 30 cm, and plastic cylinders were applied to tails to prevent climbing. The animals were tested in pairs, using cabinets preventing visual contact between them. The time spent immobile (passive hanging with no signs of struggle to escape) during a whole 6 min was scored by at least two experimenters blind to the treatment with the aid of self-developed software (Supplementary Material 3) and used as an index of despair.

#### Forced Swim Test

Approx. 24 h after TST, a complementary test for despair was conducted. The mice were placed in plastic cylinders (10 cm diameter, 25 cm height) filled with water (24 ± 1 °C) to a level preventing touching the bottom of the cylinder with tails (12 cm). The animals were tested in pairs, using the same cabinets as in TST in order to prevent observation. Each mouse was recorded for 6 min. After completion of the test, the mice were dried with paper towels and returned to their home cages for rest. Immobility during the last 4 min of the test was scored by at least two experimenters blind to treatment conditions with the aid of self-developed software (Supplementary Material 3). This value was used as an index of despair.

#### Sucrose Preference Test

Anhedonia, a second marker of depression, was measured using the sucrose preference test. At the end of the testing day 1 (approx. 56 h after the withdrawal), each cage was equipped with two identical bottles containing 2% sucrose solution in order to habituate animals to the sweet taste. After completion of experiments on the testing day 2, mice were transferred to individual cages containing one bottle with tap water and a second bottle with 2% sucrose solution. The position of the bottles was switched every 12 h and consumption of liquids was recorded for the next 64 h. Sucrose preference, which is negatively correlated to anhedonia, was calculated using the following equation:

$$\frac{\mathrm{Sucrose\ consumption }(\mathrm{g})}{\mathrm{Sucrose\ consumption }\left(\mathrm{g}\right)+\mathrm{ Water\ consumption }\left(\mathrm{g}\right)} \times 100\%$$

#### Measurement of Motor Coordination

Motor coordination was measured in order to detect behavioral signs of a possible dopaminergic toxicity after prolonged treatment with drugs stimulating DA circuitry in the brain. The procedure was conducted as previously described (Wojcieszak et al. 2020) on four consecutive days (testing days 1–4), always as the last behavioral test during the day, and after a minimum of 1 h rest from the previous tests. The test was conducted by placing mice on a rotating horizontal rod (3 cm in diameter, 5.5 cm in width; Rotarod, model RTD-4, Ataner, Lublin, Poland) made of non-slippery, sandy polyvinyl chloride that could not be gripped by the animals. The start speed was set at 4 rpm. After initial 10 s, acceleration of 20 rpm/min was applied, with the max. speed set to 40 rpm. A latency to fall was automatically recorded as a measure of motor coordination. Each mouse had three attempts daily, separated by at least 5 min rest in the home cage.

## Statistical Analysis

All analyses were performed using GraphPad Prism 6 (GraphPad Software, San Diego, CA, USA). When single main effects were analyzed, one-way ANOVA was performed, followed by post hoc tests when *p* < 0.05. When two factors were involved (time × treatment), two-way repeated measures ANOVA followed with post hoc tests (when *p* < 0.05) was performed. Details specific for each analysis are provided in figure captions.

## Results

### Experiment 1

#### 4-CMC

**Fig. 2:**
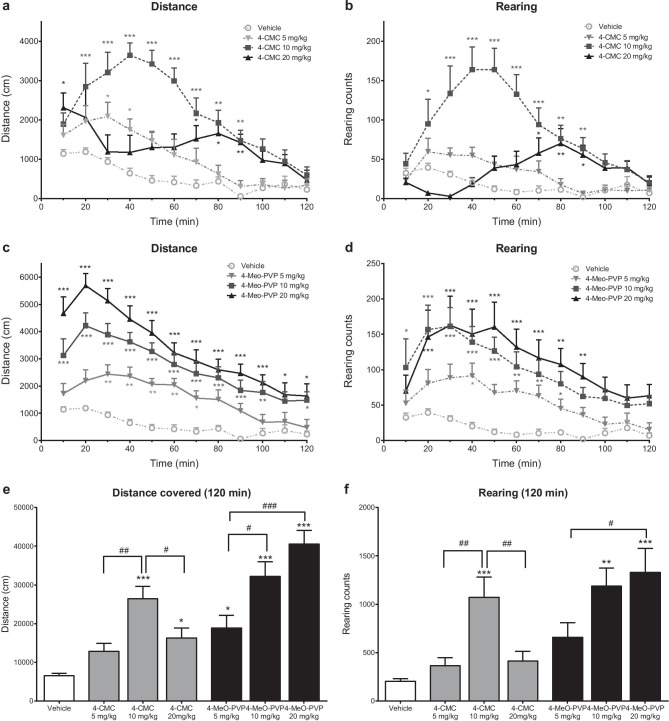
Effects of 4-CMC (5, 10, 20 mg/kg) and 4-MeO-PVP (5, 10, 20 mg/kg) on spontaneous locomotor activity of mice. Average horizontal **a**, **c** and vertical **b**, **d** activities in 10-min bins. Data are presented as mean ± standard error of the mean (SEM) (*n* = 7–8). ***p < 0.001; **p < 0.01; *p < 0.05 vs. control group; two-way repeated measures ANOVA, Dunnett’s post hoc test. Total distance traveled during 120 min **e**. Total rearing counts during 120 min **f**. Data are presented as mean ± standard error of the mean (SEM) (*n* = 7–8). ***p < 0.001; **p < 0.01; *p < 0.05 vs. control group; ###*p* < 0.001; ##*p* < 0.01; #*p* < 0.05 between indicated groups; one-way ANOVA, Tukey’s post hoc test

4-CMC caused a dose-dependent significant increase of horizontal spontaneous locomotor activity of DBA/2J mice with treatment (*F*_3,27_ = 12.88; *p* < 0.0001), time (*F*_11,297_ = 19.54; *p* < 0.0001), and treatment × time (*F*_33,297_ = 3.913; *p* < 0.0001) being significant factors. During the 20–40 (5 mg/kg), 10–90 (10 mg/kg), 0–10 and 60–90 (20 mg/kg) min post injection intervals, the distance covered during 10-min bins was higher compared with controls. The total distance covered during 120 min was significantly higher compared with controls after treatment with 10 mg/kg and 20 mg/kg 4-CMC. The 10-mg/kg dose produced the most pronounced effect (Fig. 2a, e). Noteworthy, in some mice from the 4-CMC 20-mg/kg group, pronounced stereotypies were detected (Supplementary Material 1). 4-CMC also produced a marked increase of vertical locomotor activity with significant effects of treatment (*F*_3,27_ = 9.271; *p* = 0.0002), time (*F*_11,297_ = 11.60; *p* < 0.0001), and treatment × time interaction (*F*_33,297_ = 8.603; *p* < 0.0001). A significant increase of rearing counts within 10-min bins was observed during 10–90 (10 mg/kg) and 60–90 (20 mg/kg) min post injection intervals. However, a significant increase of the total number of rearings during 120 min was observed only after treatment with 10 mg/kg of 4-CMC (Fig. 2b, f).

#### 4-MeO-PVP

4-MeO-PVP caused a significant, dose-dependent increase of horizontal spontaneous locomotor activity among the DBA/2J mice. This effect was significantly influenced by treatment (*F*_3,28_ = 23.30; *p* < 0.0001), time (*F*_11,308_ = 47.41; *p* < 0.0001), and time × treatment interaction (*F*_33,308_ = 4.386; *p* < 0.0001). Within 10-min bins, the locomotor activity was significantly increased compared with controls during the following time post injection intervals: 20–70 min (5 mg/kg), 0–100 and 110–120 min (10 mg/kg), and 0–120 min (20 mg/kg). The mice treated with 4-MeO-PVP at all tested doses covered significantly greater total distance during 120 min compared with controls, while effects of 10 mg/kg and 20 mg/kg were significantly more pronounced than 5 mg/kg (Fig. 2c, e). 4-MeO-PVP also produced a significant, dose-dependent increase of spontaneous vertical locomotor activity of DBA/2J mice, with the significant effect of treatment (*F*_3,28_ = 8.900; *p* = 0.0003), time (*F*_11,308_ = 15.47; *p* < 0.0001), and treatment × time interaction (*F*_33,308_ = 1.744; *p* = 0.0087). Within 10-min bins, significantly higher numbers of rearings were observed compared with controls during the following time post injection intervals: 30–40 min (5 mg/kg), 0–80 min (10 mg/kg), and 10–90 min (20 mg/kg). Mice treated with 10 mg/kg and 20 mg/kg of 4-MeO-PVP demonstrated a higher total number of rearings during 120 min than controls, while rearing count after 20 mg/kg was also higher than in the 5-mg/kg group (Fig. 2d, f).

### Experiment 2

**Fig. 3:**
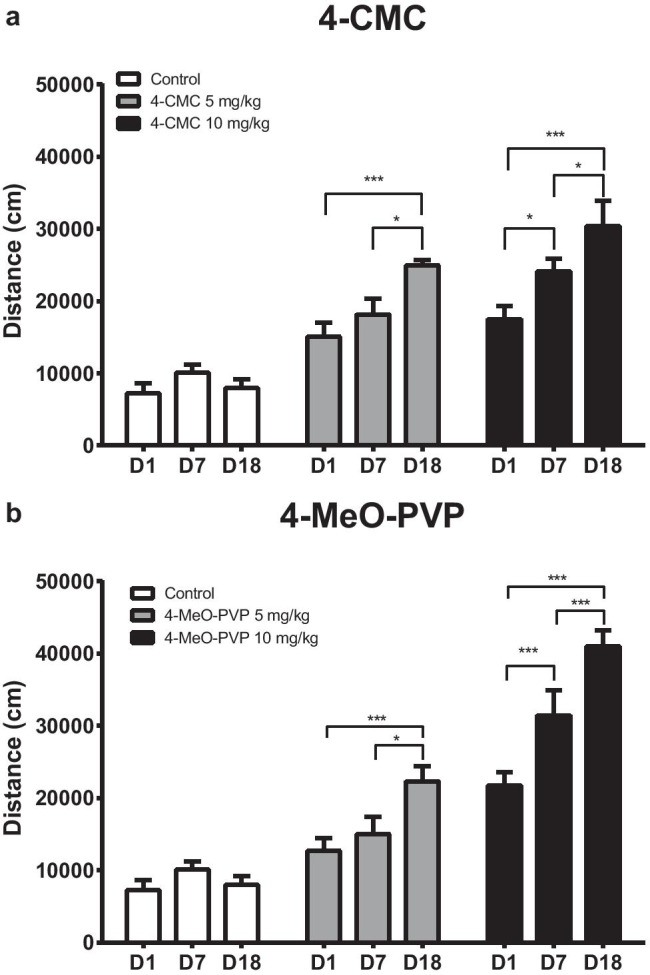
Behavioral sensitization in response to 4-CMC (5 and 10 mg/kg, **a**) and 4-MeO-PVP (5 and 10 mg/kg, **b**). Data are presented as total distance covered during 120 min and expressed as mean ± standard error of the mean (SEM) (*n* = 8). ***p < 0.001; *p < 0.05 between indicated groups; two-way repeated measures ANOVA, Tukey’s post hoc test

Both 4-CMC and 4-MeO-PVP produced significant behavioral sensitization in DBA/2J mice at 5 mg/kg and 10 mg/kg (Fig. 3).

#### 4-CMC

Repeated measures two-way ANOVA revealed that a total distance covered by mice in response to intermittent treatment with 4-CMC was significantly affected by treatment (*F*_2,21_ = 35.17; *p* < 0.0001), day (*F*_2,42_ = 16.55; *p* < 0.0001), and treatment × day interaction (*F*_4,42_ = 4.142; *p* = 0.0064). Mice treated with 5 mg/kg of 4-CMC exhibited significant sensitization to the drug’s action after 10-day withdrawal, as the response on the challenge day (D18) was significantly more potent than those on days 1 and 7 of daily treatment. During intermittent treatment with 10 mg/kg of 4-CMC, sensitization occurred sooner, as the behavioral response to the drug was more pronounced during each consecutive sessions (Fig. 3a).

#### 4-MeO-PVP

Repeated measures two-way ANOVA revealed that the total distance covered by mice in response to intermittent treatment with 4-MeO-PVP was significantly affected by treatment (*F*_2,21_ = 58.54; *p* < 0.0001), day (*F*_2,42_ = 25.67; *P* < 0.0001), and treatment × day interaction (*F*_4,42_ = 8.241; *p* < 0.0001). The lower dose of 4-MeO-PVP (5 mg/kg) evoked behavioral sensitization only after 10-day withdrawal, as the distance covered on day 18 was significantly higher compared with days 1 and 7. However, during treatment with 10 mg/kg of 4-MeO-PVP, a significant increase in the response to the drug was observed between each consecutive sessions (Fig. 3b).

### Experiment 3

#### 4-CMC

**Fig. 4:**
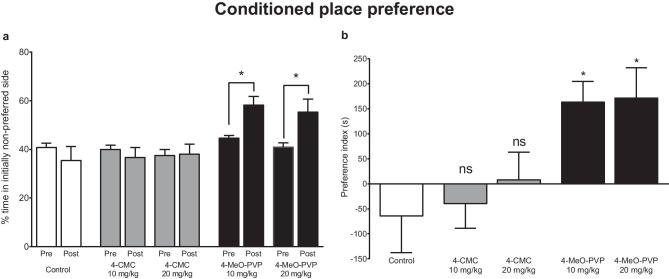
Conditioned place preference after 4-day (2 conditioning sessions daily) treatment with 4-CMC (10 mg/kg; 20 mg/kg), 4-MeO-PVP (10 mg/kg; 20 mg/kg), or saline. Time spent in initially non-preferred side during pre-test and post-test **a**. Data are presented as mean ± standard error of the mean (SEM) (*n* = 9–10). *p < 0.05 between pre-test and post-test; two-way repeated measures ANOVA, Bonferroni’s post hoc test. Preference index (time spent in initially non-preferred side: post-test–pre-test) **b**. *p < 0.05 vs. control group, one-way ANOVA, Dunnett’s *post hoc* test

In mice treated with 4-CMC, the time spent in the initially non-preferred side was not affected by treatment (*F*_2,27_ = 0.01619; *p* = 0.9840), day (*F*_1,27_ = 0.8273; *p* = 0.3711), and treatment × day interaction (*F*_2,27_ = 0.3470; *p* = 0.7099) (Fig. 4a). Moreover, a preference index of mice treated with 4-CMC at doses of 10 mg/kg and 20 mg/kg did not differ from control animals (*F*_2,27_ = 0.3686; *p* = 0.6952) (Fig. 4b).

#### 4-MeO-PVP

Repeated measures two-way ANOVA indicated that the time spent in the initially non-preferred side by 4-MeO-PVP-treated mice was significantly affected by treatment (*F*_2,26_ = 6.811; *p* = 0.0042), day (*F*_1,26_ = 7.115; *p* = 0.0130), and treatment × day interaction (*F*_2,26_ = 5.023; *p* = 0.0143). The time spent in the initially non-preferred side changed significantly between pre-test and post-test in 4-MeO-PVP 10-mg/kg and 20-mg/kg groups (Fig. 4a). These results were further confirmed by a subsequent one-way ANOVA of preference indexes of 4-MeO-PVP-treated mice (*F*_2,26_ = 5.039; *p* = 0.0141) followed Dunnett’s post hoc test indicating a significant difference between both 4-MeO-PVP groups vs. control (Fig. 4b).

### Experiment 4

#### Body Mass

All mice were weighed daily. No relevant changes in body mass (> 1.0 g) were observed within the treatment period for any treatment group (data not shown).

#### Spontaneous Locomotor Activity

**Fig. 5:**
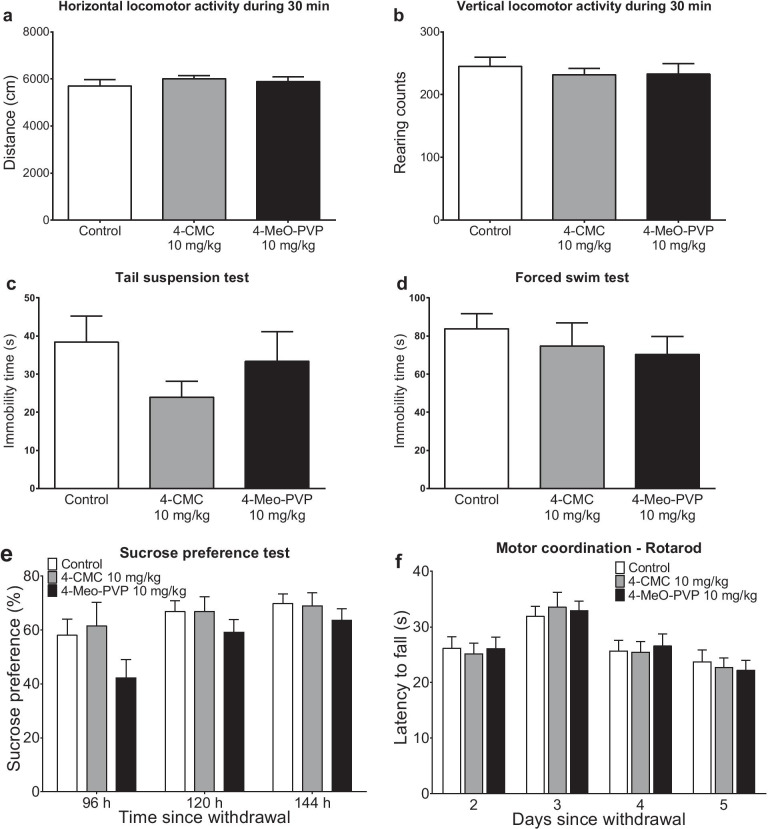
Effects of withdrawal of 4-CMC (10 mg/kg) and 4-MeO-PVP (10 mg/kg) after 14-day treatment. Spontaneous horizontal **a** and vertical **b** locomotor activities during 30 min. Immobility times in tail suspension test **c** and forced swim test **d**. Data are presented as mean ± standard error of the mean (SEM) (*n* = 12), ordinary one-way ANOVA. Sucrose preference **e**. Motor coordination measured with Rotarod **f**. Data are presented as mean ± standard error of the mean (SEM) (*n* = 12), two-way repeated measures ANOVA

Ordinary one-way ANOVA did not indicate that 48-h withdrawal from 14-day treatment with either 4-CMC (10 mg/kg) or 4-MeO-PVP (10 mg/kg) had any effect on either horizontal (*F*_2,33_ = 0.5427; *p* = 0.5863) or vertical (*F*_2,33_ = 0.2626; *p* = 0.7707) locomotor activities of mice during 30-min session in a drug-free state (Fig. 5a, b).

#### Depression Markers

Withdrawal from 4-CMC (10 mg/kg) and 4-MeO-PVP (10 mg/kg) did not affect the immobility time, serving as an index of despair, in the tail suspension test 2 days after cessation (one-way ANOVA: *F*_2,33_ = 1.289; *p* = 0.2892), and in the forced swim test 3 days after the discontinuation of the treatment (one-way ANOVA: *F*_2,33_ = 0.4734; *p* = 0.6270) (Fig. 5c, d).

Moreover, there was no significant main effect of treatment (*F*_2,33_ = 1.256; *p* = 0.2981) on the cumulative sucrose preference during 80–144 h after the withdrawal period (Fig. 5e).

#### Motor Coordination

Two-way repeated measures ANOVA revealed that treatment (*F*_2,33_ = 0.005319; *p* = 0.9947) and treatment × time interaction (*F*_6,99_ = 0.3161; *p* = 0.9272) had no significant main effect on the motor performance of DBA/2J mice (Fig. 5f).

## Discussion

The locomotor response of rodents is commonly assessed in order to predict the psychostimulant effects of drugs of abuse, these being related to increased extracellular levels of dopamine in the structures of the central nervous system, such as the nucleus accumbens and striatum. The present study demonstrates that 4-CMC and 4-MeO-PVP produce a dose-dependent increase of horizontal locomotor activity in DBA/2J mice. Interestingly, the locomotor stimulation evoked by 4-CMC was less pronounced at 20 mg/kg compared with 10 mg/kg, because of a slower onset, presumably associated with an incidence of stereotypies in some mice during the initial 60 min after treatment (Supplementary Material 1). Our results are in line with previously reported locomotor stimulation observed in Swiss-Webster mice (Gatch et al. 2019) treated with 4-CMC (2.5 and 5 mg/kg), where the similar pattern of locomotor stimulation, with a slow onset and long duration, was observed after treatment with 4-CMC and MDMA. Interestingly, in the current study, the onset of locomotor stimulation induced by 4-CMC at the highest tested dose of 20 mg/kg was also significantly delayed, similarly to MDMA (50 mg/kg) reported by Gatch and coworkers (2019).

The second experiment aimed to assess whether 4-CMC and 4-MeO-PVP could produce behavioral sensitization, defined as an augmented psychomotor response to the drug that can be observed after its re-administration following withdrawal from repeated exposure (Macúchová and Šlamberová 2017). Sensitization is known to occur after prolonged treatment with various psychostimulants and has been proposed as a symptom of an initial stage of psychostimulant addiction as it contributes to the drug craving (Duart-Castells et al. 2019). Here, we demonstrate that both 4-CMC and 4-MeO-PVP produce significant locomotor sensitization that can be observed after 7 days of daily treatment (both drugs at 10 mg/kg) and during the challenge conducted 10 days after withdrawal from intermittent treatment (both drugs at 5 and 10 mg/kg). These results are in line with other published data on properties of synthetic cathinones. Behavioral sensitization occurs in laboratory rodents in response to MDPV, mephedrone, and methylone after 6 or 7 days of treatment (Allen et al. 2019; Berquist et al. 2016; Gregg et al. 2013; Kohler et al. 2018).

Our results also demonstrate that 4-MeO-PVP (10 and 20 mg/kg) produces significant CPP after four drug-paired sessions in mice. However, no such effect was observed for 4-CMC used at both doses that produced significant increases of locomotor activity during 120 min. Results of our study demonstrating that 4-MeO-PVP produces CPP in mice are in line with findings showing that other synthetic cathinones, e.g., MDPV—highly selective for DAT (Eshleman et al. 2013), evoke similar effects in both rats (Atehortua-Martinez et al. 2019; Gregg et al. 2016; King et al. 2015; Oliver et al. 2018) and mice (Karlsson et al. 2014). The place preference behavior was also induced by other pyrrolidine-containing cathinones, i.e., α-PVT, α-PVP, α-PBP (Cheong et al. 2017; Gatch et al. 2015). A possible explanation of the distinct effects of 4-CMC and 4-MeO-PVP in the CPP experiment is the difference in their molecular mechanisms of action, as 4-MeO-PVP has a DAT/SERT inhibition ratio of 37.1, compared with 3.22 for 4-CMC. Additionally, 4-CMC, in contrast to 4-MeO-PVP, is also a potent releaser of DA and 5-HT (Bonano et al. 2015; Eshleman et al. 2017). Our results are in line with those indicating that compounds with increased activity at SERT relative to DAT are less robustly self-administered by animals (Iversen et al. 2014); they also agree with the general notion that cathinones with more pronounced action toward SERT are more likely to act as empathogens with a lower abuse potential, compared with methamphetamine-like psychostimulants with substantial DAT preference (Liechti 2015; Zawilska and Wojcieszak 2013). On the other hand, the fact that 4-CMC (10 and 20 mg/kg) did not appear to display rewarding effects does not definitively mean that this drug is devoid of abuse potential, as it has produced rewarding effects at 10 mg/kg in intracranial self-stimulation paradigm in rats (Bonano et al. 2015).

Finally, we attempted to examine whether behavioral markers of depression could occur in mice during withdrawal from prolonged, 14-day exposure to 4-CMC (10 mg/kg) or 4-MeO-PVP (10 mg/kg). To do so, we utilized the battery of validated and broadly used tests measuring two major signs of depression, namely despair (tail suspension test and forced swim test) and anhedonia (sucrose preference test). First, treatment with either 4-CMC or 4-MeO-PVP for 14 days did not induce general toxicity: no changes were observed in the body mass of the treated mice, nor in the non-stimulated spontaneous locomotor activity. All three behavioral tests measuring markers of depression consistently demonstrated the lack of significant increase of despair and anhedonia during 4 days, starting 48 h after withdrawal of the drug. These results are in contrast to previous findings on enhanced depression markers after withdrawal from MDPV in rodents. Philogene-Khalid and colleagues (2017) observed increased immobility in rats in the forced swim test 48 h after 10-day binge treatment with MDPV (3 × 1 mg/kg, 1 h apart). In another study, sucrose preference was decreased in rats 48 h after both acute and binge treatment with MDPV (3 mg/kg) (Atehortua-Martinez et al. 2019). One possible explanation of this disparity might be that MDPV has a much higher DAT/SERT ratio (3,4-MDPV: 110; 4-MeO-PVP: 37.1; 4-CMC: 3.22) and DAT inhibition potency (IC_50_ MDPV: 0.0126 µM; 4-MeO-PVP: 0.1126 µM; 4-CMC: 0.208 µM) than 4-CMC and 4-MeO-PVP (Eshleman et al. 2013, 2017). Therefore, it could be speculated that the ability of the drug to induce the depressive state after withdrawal, resulting from adaptive changes in monoaminergic neurotransmission, may depend on its potency and selectivity towards DAT. In addition, discontinuation of a binge mephedrone in Swiss CD-1 mice resulted in an increase of immobility in the forced swim test (Martínez-Clemente et al. 2014), which may suggest that binge treatment has a greater capacity to induce depressive effects after withdrawal, due to the prolonged exposure to very high drug concentrations, leading to monoamine depletion. On the other hand, in line with the current study, depressive changes in behavior were not found using the tail suspension test in C57BL/6J mice after withdrawal from a high dose (30 mg/kg) 4-day binge treatment with mephedrone and methylone (den Hollander et al. 2013).

Conflicting reports on the ability of synthetic cathinones to produce signs of depression after cessation are not surprising, since there are also contrasting data on the pro-depressive effects of amphetamine withdrawal in rodents. Cryan and colleagues (2003) report a significant increase of depression-related markers in rats withdrawn from the 6-day treatment with amphetamine, measured as an elevation of rewarding thresholds in intracranial self-stimulation paradigm and increased immobility time in the forced swim test. Moreover, the authors observe an increased immobility time in the tail suspension test in DBA/2Ha mice 24 h post withdrawal from 7-day treatment with amphetamine. On the other hand, Russig and colleagues (2003) found no signs of depression in rats after withdrawal from 6-day treatment with amphetamine in both escalating dose and intermittent dose schedules, using the forced swim test, the learned helplessness assay, and operant responding for sucrose on a progressive ratio schedule.

We also examined the motor coordination of mice in order to detect behavioral signs of impairment of dopaminergic circuitry, which could appear after the prolonged exposure to drugs enhancing DA neurotransmission within CNS. We found no significant decreases of motor performance of mice on the rotarod, suggesting that 14-day treatment with 4-CMC (10 mg/kg) or 4-MeO-PVP (10 mg/kg) did not produce severe impairment of dopaminergic structures, which would be manifested in the behavioral assay.

We would like to emphasize that lack of significant effects on depressive markers found in a particular study should not be taken as a proof that a drug would not produce pro-depressive withdrawal effects in humans. There are multiple experimental factors that could obscure these effects, such as the length and type (binge vs. intermittent) of treatment, withdrawal period until behavioral testing, types of behavioral tests performed, and species and strain of laboratory animals used. Therefore, to obtain relevant results, the battery of behavioral test should be used, and preferably, results should be reproduced using different strains/species of laboratory animals.

## Conclusions

This study demonstrates that 4-CMC and 4-MeO-PVP produce dose-dependent increases in horizontal and vertical activities in DBA/2J mice. Both drugs induce behavioral sensitization after intermittent treatment with 5- and 10-mg/kg doses. 4-MeO-PVP (10 and 20 mg/kg) is endowed with marked rewarding properties as it produces conditioned place preference, while this effect is absent after 4-CMC (10 and 20 mg/kg) administration, a phenomenon that could be related to the stronger selectivity of 4-MeO-PVP towards the dopamine transporter compared with 4-CMC. Finally, both 4-CMC (10 mg/kg) and 4-MeO-PVP (10 mg/kg) do not produce significant depression-related behaviors after withdrawal from the 14-day treatment.

## Supplementary Information

Below is the link to the electronic supplementary material.

Supplementary file 1 (PDF 55 KB)

CPP software (ZIP 2 MB)

Despair software (ZIP 2 MB)

Instructions for Despair and CPP by Jakub Wojcieszak (DOCX 12 KB)
