# Supplementary material for: Behavioral Effects of 4-CMC and 4-MeO-PVP in DBA/2J Mice After Acute and Intermittent Administration and Following Withdrawal from Intermittent 14-Day Treatment
Source: Neurotox Res. 2021 Jan 11;39(3):575–87. doi: 10.1007/s12640-021-00329-x (PMC8096775; doi:10.1007/s12640-021-00329-x)
Supplement: Supplementary file 1 — (PDF 55 KB) [file 12640_2021_329_MOESM1_ESM.pdf]

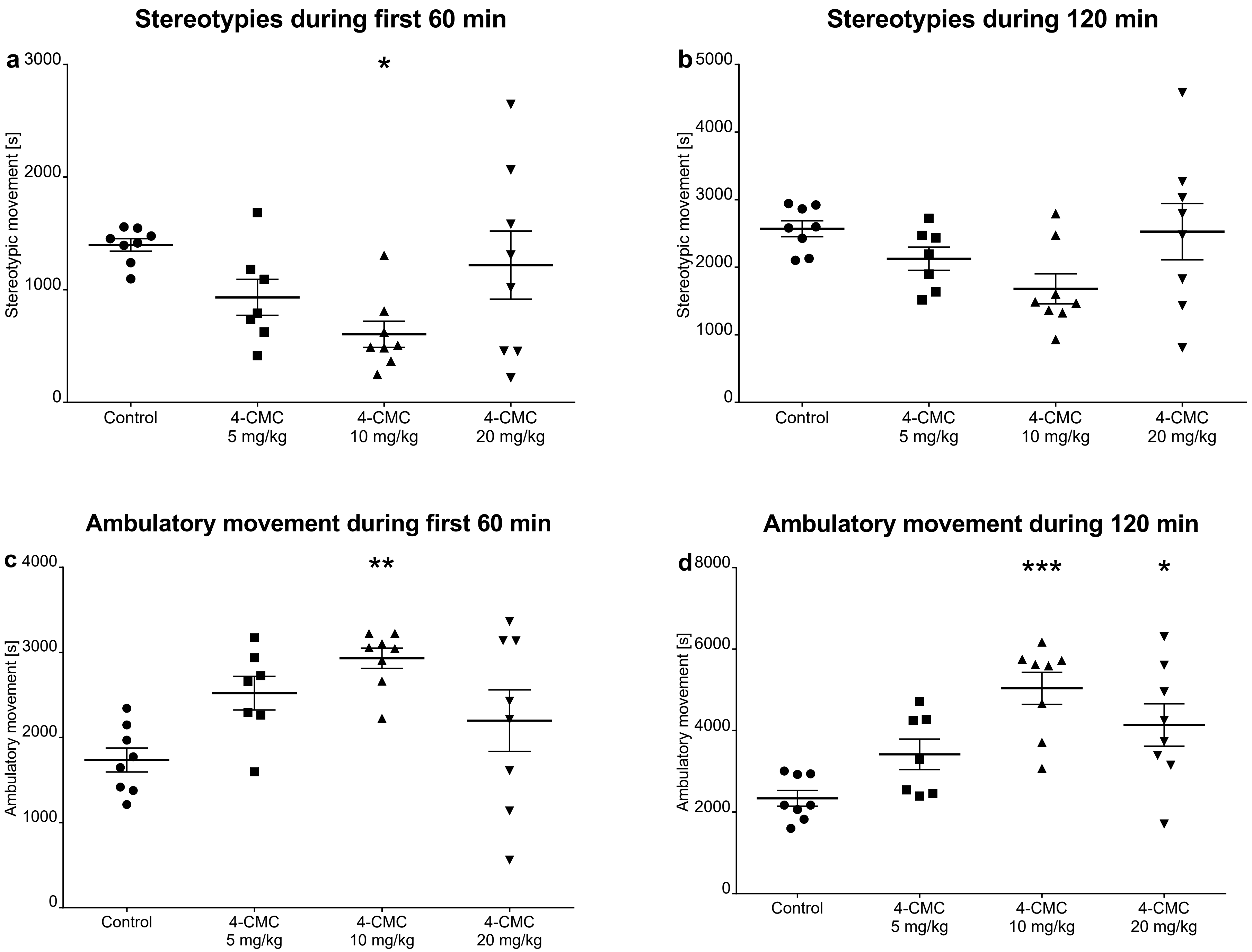

Time of stereotypic (panels a, b) and ambulatory movement (panels c, d) during first 60 min (panels a, c) or whole 120 min (panels b, d) of mice treated with saline or 4-CMC (5, 10 and 20 mg/kg). Each symbol represents one mouse. Horizontal lines represent mean ± SEM. N = 7-8. *** $p < 0.001$; ** $p < 0.01$; * $p < 0.05$ vs. control (one-way ANOVA, Tukey's post hoc test).
