# Supplementary material for: Behavioral Effects of 4-CMC and 4-MeO-PVP in DBA/2J Mice After Acute and Intermittent Administration and Following Withdrawal from Intermittent 14-Day Treatment
Source: Neurotox Res. 2021 Jan 11;39(3):575–87. doi: 10.1007/s12640-021-00329-x (PMC8096775; doi:10.1007/s12640-021-00329-x)
Supplement: Supplementary file 4 — Instructions for Despair and CPP by Jakub Wojcieszak (DOCX 12 KB) [file 12640_2021_329_MOESM4_ESM.docx]

**Instructions for Despair and CPP by Jakub Wojcieszak**

Despair and CPP support video files compatible with Windows Media Player, such as .wmv or .avi

**Despair**

1. This software allows to manually score one animal at the time.
2. To blind the experimenter we name files as random numbers. Then, at the end of video, when the animal is scored, we show a label indicating all experimental details to the camera.
3. Click on “Select” to choose a file for analysis.
4. Click on “Start” when animal is ready to be scored.
5. When the animal is immobile press and hold space bar.
6. When the assessment time is over click on “End” to reveal the results.
   1. The immobility score is hidden during the assessment to prevent confirmation bias.
7. Buttons with time labels on the left allow the user to go back to the indicated time of measurement in the case of error/distraction. Immobility score will be rolled back.

**CPP**

1. This software allows to manually score two animals at the time.
2. To blind the experimenter we name files as random numbers. Then, at the end of video, when the animal is scored, we show a label indicating all experimental details to the camera.
3. Click on “Select” to choose a file for analysis.
4. Click on the respective “Start L” or “Start R” when animal is ready to be scored.
5. Change between “Black” and “Stripes” by clicking on the switches or by pressing “Z” (left) or “X” (right).
6. The program shows results for two commonly used times in CPP experiments: 15 and 20 minutes.
7. Buttons with time labels on the bottom allow the user to go back to the indicated time of measurement in the case of error/distraction. Immobility score will be rolled back.
